# An unusual histological finding following myomectomy

**DOI:** 10.52054/FVVO.14.4.049

**Published:** 2023-01-27

**Authors:** F Macdonald, A Okojie, A Gayen, R Mallick

**Affiliations:** University Hospitals Sussex NHS Foundation Trust, Princess Royal Hospital, Lewes Road, United Kingdom RH16 4EX

**Keywords:** Schwannoma, broad ligament fibroid, pelvic mass

## Abstract

Ancient schwannomas are benign tumours arising from the neural sheath of peripheral, cranial and autonomic nerves. They are commonly situated in the inner ear and spine with pelvic manifestations being rare. We present the case of a 30-year-old patient, who presented with an abdominal mass. MRI imaging suggested a broad ligament fibroid and open surgery was undertaken to remove it. Subsequent histology confirmed an ancient schwannoma. This case report details the rarity of such a condition and the need for a high index of suspicion as well as outlining management options and surveillance.

## Introduction

Schwannomas are usually benign tumours which develop from the Schwann cells surrounding nerve cells. Those located in the head and neck account for 45% of schwannomas: other common sites include the mediastinum and spine. The majority of these tumours are sporadic but can be associated with genetic conditions such as neurofibromatosis 1 and 2.

Pelvic schwannomas are often asymptomatic and may be diagnosed incidentally. When they do present with symptoms, it is commonly due to the mass effect of the tumour on surrounding structures but can be vague, such as abdominal distension or heaviness, urinary frequency, and constipation. It is often difficult to determine the diagnosis pre- operatively, but it is important to use imaging to determine the size, location, and interaction with local structures to aid with operative planning.

While broad ligament fibroids are not an unusual gynaecological finding, schwannomas in the pelvis are rare, accounting for 1-3% of all cases (Borghese et al., 2002). Schwannomas arising from the broad ligament are even more scarce, with less than a handful reported in the wider literature. The diagnosis of this type of tumour is difficult to make pre-operatively as there are often no pathognomonic features. This case report presents a patient who underwent an open myomectomy for a suspected broad ligament fibroid, which was subsequently found to be an ancient schwannoma.

## Case report

A 30-year-old patient was referred to gynaecology with a history of worsening abdominal swelling, pressure symptoms and a known uterine fibroid. Magnetic resonance imaging (MRI) revealed an 11cm likely broad ligament fibroid and both medical and surgical treatment options were discussed. Initial medical treatment with ulipristal acetate was largely ineffective so the patient opted for surgery. A myomectomy was discussed in detail including both the laparoscopic approach (and morcellation for specimen retrieval) and an open procedure. The decision was made to make the final decision intraoperatively depending on the size and mobility of the fibroid. As the fibroid was immobile and fixed within the pelvis on both vaginal and abdominal examination following general anaesthetic, the decision was to proceed with an open myomectomy.

A lower umbilical midline incision was utilised, and findings were of a 12cm fibroid arising wholly within the broad ligament with the ureter lying inferolateral. No obvious connection was seen to the uterus and no other fibroids were visible. Monopolar electrocautery (50W cut, 50W coagulation) was utilised to open the broad ligament and a full right ureterolysis was undertaken to lateralise the ureter.

Following ureterolysis, the suspected fibroid was enucleated using a myoma screw and a combination of blunt and sharp dissection. The macroscopic appearances of the mass and its behaviour during dissection were in keeping with a fibroid. The specimen was sent for histology and a routine abdominal mass closure was performed. Blood loss was 350ml and the patient made a good recovery post-operatively.

The histopathology subsequently revealed an ancient schwannoma, and no malignancy was seen in the sections examined. The neoplasm showed well defined margins with degenerative cystic change and haemorrhage present.

A neurology review was subsequently arranged which excluded any other schwannomas or associated genetic disorders. There was no family history of schwannomas or neuromas, or personal history of café au lait spots or hearing impairment. The neurologist proposed that it was likely the patient had a somatic mutation leading to a schwannoma causing unilateral segmental fibromatosis. The management for this included MRI of the chest, head and neck and genetic testing for neurofibromatosis followed up by pelvic surveillance every 5 years. From a gynaecology perspective, the patient recovered well with no concerns at follow-up and has had a successful pregnancy.

**Figure 1:**
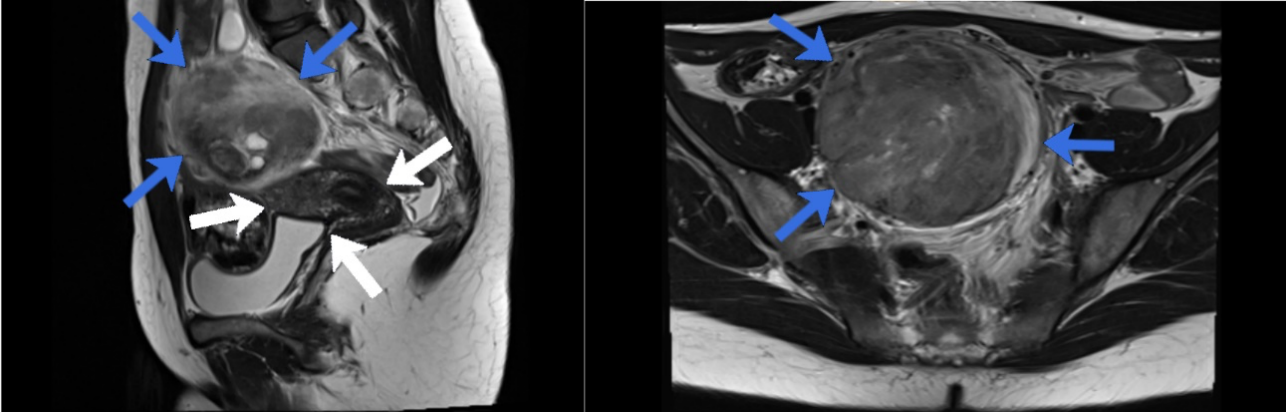
MRI images in T2W: A: MRI in sagittal view showing the uterus (white arrows) and the schwannoma (blue arrows). B: MRI in axial view showing the schwannoma (blue arrows).

**Figure 2:**
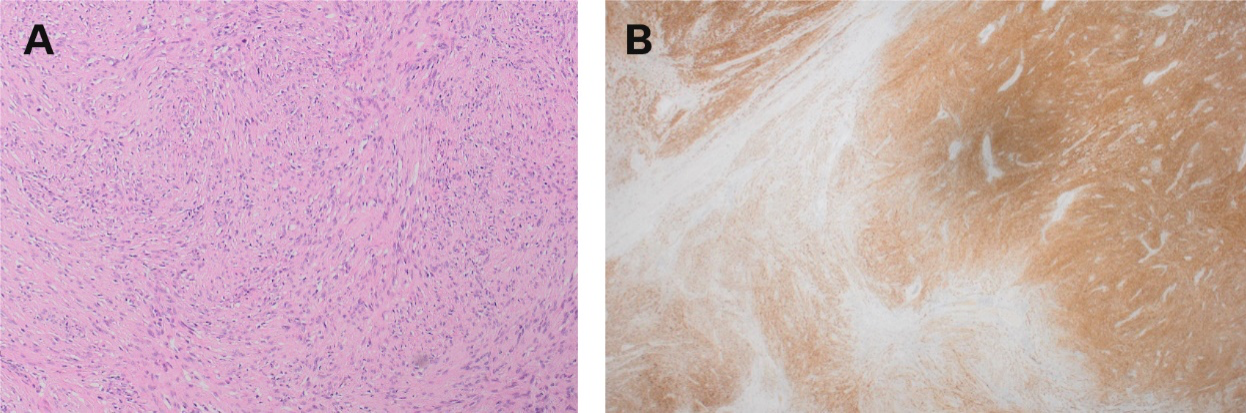
Histology images: A: Lesional cells are narrow, elongated, wavy with tapered ends interspersed with collagen fibres. B: Immunohistochemistry shows diffuse positive staining with S100 protein.

## Discussion

Schwannomas are rare, benign, usually solitary, neural tumours which arise from the neural sheath; they are well circumscribed with a capsule derived from the epineurium. They are easily dissected from adjacent tissues, making laparoscopic resection possible whilst mimicking the appearance of a fibroid capsule. Once resected, the recurrence rates of a schwannoma are low and if it does recur it is likely that the resection was not radical (Borghese et al., 2002).

Ancient schwannomas are a rare variant of schwannoma. It has distinctive features seen on histology due to their slow growing nature: the large mass leads to vascular insufficiency leading to degenerative changes including cystic necrosis, fibrosis, calcification, and degenerative nuclei (Choudry et al., 2009).

Epidemiology suggests an incidence of 1.1 per 100,000. Affected sites include the spine, inner ear, gastro-intestinal tract, and mediastinum: most commonly affecting the inner ear. There is a predilection in the second and third decades of life and they are more common in women (Wong et al., 2010). Pelvic sites are rare but have been reported in the non-pregnant and pregnant state (Nithya et al., 2017).

Schwannomas can be sporadic or occur in association with genetic conditions such as Carney complex, neurofibromatosis and schwannomatosis and tend to be multiple in the latter two conditions. Schwannomas are slow growing, with rare malignant transformation (<1%).

Schwannomas of the female genitalia are scarce. Wider literature reports 63 schwannomas arising from the female genital tract (Jiang et al., 2016) and they have been reported in the vagina (9%) (Ellison et al., 1992), vulva (27%) (Das et al., 2008), cervix (24%) (Dey et al., 2018), and pelvis (Crist et al., 2017). Pelvic schwannomas, especially those arising from the broad ligament, are extremely rare; they often arise from the hypogastric or sacral plexus. Presentation is usually related to the size of the mass or secondary to its compressive effects.

Imaging with MRI helps to demarcate the lesion; they appear as a clearly delineated round mass with low signal intensity on T1, heterogeneously hyperintense on T2, and intense enhancement on contrast MRI. Uterine fibroids, a differential diagnosis, on MRI characteristically have high signal intensity on T1 and low signal intensity on T2. However, fibroids with degeneration have similar appearance radiologically (Takeuchi et al., 2008). The use of ultrasound for pre-operative diagnosis has not been shown to be useful in distinguishing schwannoma from its differential diagnoses. Li et al., 2007 in a retrospective review of 82 retroperitoneal schwannomas, highlighted that only 15.9% were identified pre-operatively. Thus, the diagnosis of schwannomas with imaging is challenging given the lack of definitive criteria: it has been recommended to use surgical excision and histological examination to confirm the diagnosis. Percutaneous biopsy can be undertaken, if the diagnosis is suspected, with accuracy rates of up to 98% and 81% in distinguishing benign from malignant soft tissue types and identifying benign soft tissue tumour subtypes, respectively (Strauss et al., 2011). However, access to retroperitoneal lesions can be challenging and results inaccurate for large and mixed lesions (Di Furia et al., 2018).

Treatment is complete excision and as the majority are benign, enucleation is usually curative. Being neurological in origin, if suspected pre-operatively, the patient should be counselled that neurological deficits may occur depending on the location of the pathology. Nedelcu et al. (2013) reported a 44% risk of neurological sequelae in the laparoscopic removal of neurological retro-rectal masses, however data is sparse and appears to be directly related to mass position. Attention to surrounding structures, meticulous blunt dissection and enucleation appear to be key to a successful removal (Di Furia et al., 2018). Long-term prognosis is good, however follow up is important as surgical excision may be incomplete. Recurrence rates with incomplete resection have been reported between 16-54% (Okuyama et al., 2014).

## Conclusion

Pelvic schwannomas are rare, especially those in the broad ligament with very few documented in the wider literature. Diagnosis is usually made histologically as imaging is often inconclusive. A high index of suspicion is needed, especially if risk factors are present and percutaneous biopsy pre- operatively to confirm diagnosis and aid surgical planning may be helpful. Surgical excision and complete resection are recommended to reduce recurrence. Following diagnosis, neurological review is essential to exclude extra-pelvic involvement and genetic predispositions.
